# Supplementary material for: Discovery and application of insertion-deletion (INDEL) polymorphisms for QTL mapping of early life-history traits in Atlantic salmon
Source: BMC Genomics. 2010 Mar 8;11:156. doi: 10.1186/1471-2164-11-156 (PMC2838853; doi:10.1186/1471-2164-11-156)
Supplement: Additional file 2 — Information on developed 76 locus single-run INDEL panel in Atlantic salmon. Information on fluorescence labeling, primer concentrations, PCR pooling and links to alignments, INDEL motifs and GENESCAN (Burge and Karlin 1997) predictions of genes/exons are available in html format. [file 1471-2164-11-156-S2.ZIP › Additionalfile2/snpsummary2571.html]

```
Cluster 483 Contig 2

prev  Summary    Contig List  next
```

Size of Consensus sequence = 2014

Number of sequences = 38

Minimum redundancy = 6

Key

A gi|25997652|gb|CA768397.1|CA768397 ssalpitl509310 head Salmo salar cDNA, mRNA sequence  
B gi|24351893|gb|CA045723.1|CA045723 ssalpitl505258 head Salmo salar cDNA, mRNA sequence  
C gi|84973499|gb|DW471900.1|DW471900 SGP286017 Atlantic salmon Spleen cDNA library Salmo salar cDNA clone MI5-1859 5', mRNA sequence  
D gi|24464282|gb|BQ036901.1|BQ036901 SS5-0031 Atlantic Salmon spleen SSH Salmo salar cDNA clone SS5-0031, mRNA sequence  
E gi|85044971|gb|DW573149.1|DW573149 EST\_ssal\_rgb2\_37568 rgb2 Salmo salar cDNA clone ssal\_rgb2\_561\_060\_fwd 3', mRNA sequence  
F gi|24383000|gb|CA052757.1|CA052757 ssalrgb515086 mixed\_tissue Salmo salar cDNA, mRNA sequence  
G gi|45312245|gb|CK882614.1|CK882614 SGP147259 Atlantic salmon Heart cDNA library Salmo salar cDNA clone H6-0162 5', mRNA sequence  
H gi|89849313|gb|DY705436.1|DY705436 EST\_ssal\_rgb2\_61175 ssalrgb2 mixed\_tissue Salmo salar cDNA Salmo salar cDNA clone ssal\_rgb2\_598\_175\_fwd 3', mRNA sequence  
I gi|85024089|gb|DW552745.1|DW552745 EST\_ssal\_rgb2\_17164 rgb2 Salmo salar cDNA clone ssal\_rgb2\_528\_099\_fwd 3', mRNA sequence  
J gi|85027533|gb|DW556189.1|DW556189 EST\_ssal\_rgb2\_20608 rgb2 Salmo salar cDNA clone ssal\_rgb2\_533\_239\_fwd 3', mRNA sequence  
K gi|89863024|gb|DY719147.1|DY719147 EST\_ssal\_rgb2\_74886 ssalrgb2 mixed\_tissue Salmo salar cDNA Salmo salar cDNA clone ssal\_rgb2\_623\_070\_fwd 3', mRNA sequence  
L gi|89849953|gb|DY706076.1|DY706076 EST\_ssal\_rgb2\_61815 ssalrgb2 mixed\_tissue Salmo salar cDNA Salmo salar cDNA clone ssal\_rgb2\_599\_168\_fwd 3', mRNA sequence  
M gi|24346911|gb|CA044497.1|CA044497 ssalmgd001054 gut Salmo salar cDNA, mRNA sequence  
N gi|24383667|gb|CA053424.1|CA053424 ssalrga516063 mixed\_tissue Salmo salar cDNA, mRNA sequence  
O gi|85033328|gb|DW561984.1|DW561984 EST\_ssal\_rgb2\_26403 rgb2 Salmo salar cDNA clone ssal\_rgb2\_542\_342\_fwd 3', mRNA sequence  
P gi|89863970|gb|DY720093.1|DY720093 EST\_ssal\_rgb2\_75832 ssalrgb2 mixed\_tissue Salmo salar cDNA Salmo salar cDNA clone ssal\_rgb2\_624\_255\_fwd 3', mRNA sequence  
Q gi|85055070|gb|DW583248.1|DW583248 EST\_ssal\_rgb2\_47667 rgb2 Salmo salar cDNA clone ssal\_rgb2\_576\_368\_fwd 3', mRNA sequence  
R gi|117454307|gb|EG786526.1|EG786526 EST\_ssal\_evd\_7193 ssalevd thymus Salmo salar cDNA Salmo salar cDNA clone ssal\_evd\_508\_125\_rev 5', mRNA sequence  
S gi|89871807|gb|DY727930.1|DY727930 EST\_ssal\_rgb2\_83669 ssalrgb2 mixed\_tissue Salmo salar cDNA Salmo salar cDNA clone ssal\_rgb2\_637\_068\_fwd 3', mRNA sequence  
T gi|117835593|gb|EG908289.1|EG908289 EST\_ssal\_evf\_10617 ssalevf mixed\_tissue Salmo salar cDNA Salmo salar cDNA clone ssal\_evf\_512\_225\_rev 5', mRNA sequence  
U gi|83640087|gb|DW006677.1|DW006677 SGP296175 Atlantic salmon Brain cDNA library Salmo salar cDNA clone HJ4-3337 5', mRNA sequence  
V gi|117835592|gb|EG908288.1|EG908288 EST\_ssal\_evf\_10616 ssalevf mixed\_tissue Salmo salar cDNA Salmo salar cDNA clone ssal\_evf\_512\_225\_fwd 3', mRNA sequence  
W gi|85019208|gb|DW547864.1|DW547864 EST\_ssal\_rgb2\_12283 rgb2 Salmo salar cDNA clone ssal\_rgb2\_520\_265\_fwd 3', mRNA sequence  
X gi|117514152|gb|EG845911.1|EG845911 EST\_ssal\_eve\_58612 ssaleve thyroid Salmo salar cDNA Salmo salar cDNA clone ssal\_eve\_579\_338\_rev 5', mRNA sequence  
Y gi|117454308|gb|EG786527.1|EG786527 EST\_ssal\_evd\_7194 ssalevd thymus Salmo salar cDNA Salmo salar cDNA clone ssal\_evd\_508\_125\_fwd 3', mRNA sequence  
Z gi|89871806|gb|DY727929.1|DY727929 EST\_ssal\_rgb2\_83668 ssalrgb2 mixed\_tissue Salmo salar cDNA Salmo salar cDNA clone ssal\_rgb2\_637\_068\_rev 5', mRNA sequence  
a gi|117514151|gb|EG845910.1|EG845910 EST\_ssal\_eve\_58611 ssaleve thyroid Salmo salar cDNA Salmo salar cDNA clone ssal\_eve\_579\_338\_fwd 3', mRNA sequence  
b gi|89863969|gb|DY720092.1|DY720092 EST\_ssal\_rgb2\_75831 ssalrgb2 mixed\_tissue Salmo salar cDNA Salmo salar cDNA clone ssal\_rgb2\_624\_255\_rev 5', mRNA sequence  
c gi|89849954|gb|DY706077.1|DY706077 EST\_ssal\_rgb2\_61816 ssalrgb2 mixed\_tissue Salmo salar cDNA Salmo salar cDNA clone ssal\_rgb2\_599\_168\_rev 5', mRNA sequence  
d gi|89863023|gb|DY719146.1|DY719146 EST\_ssal\_rgb2\_74885 ssalrgb2 mixed\_tissue Salmo salar cDNA Salmo salar cDNA clone ssal\_rgb2\_623\_070\_rev 5', mRNA sequence  
e gi|15280947|gb|BI468069.1|BI468069 EST00479 Atlantic salmon Lambda Zap Express liver cDNA library Salmo salar cDNA clone LRR8-c08 5' similar to 146D Nuclear Protein, mRNA sequence  
f gi|15846186|gb|BG936358.1|BG936358 SS1-0730 Atlantic Salmon spleen Salmo salar cDNA clone SS1-0730 similar to Splicing factor 3b, mRNA sequence  
g gi|85019207|gb|DW547863.1|DW547863 EST\_ssal\_rgb2\_12282 rgb2 Salmo salar cDNA clone ssal\_rgb2\_520\_265\_rev 5', mRNA sequence  
h gi|85044970|gb|DW573148.1|DW573148 EST\_ssal\_rgb2\_37567 rgb2 Salmo salar cDNA clone ssal\_rgb2\_561\_060\_rev 5', mRNA sequence  
i gi|62630256|gb|CX727337.1|CX727337 EST00018 SSH1 Salmo salar cDNA similar to splicing factor 3b, subunit 1, mRNA sequence  
j gi|57125505|gb|CX356946.1|CX356946 ssalrgb515086\_rev\_0 mixed\_tissue Salmo salar cDNA, mRNA sequence  
k gi|85024088|gb|DW552744.1|DW552744 EST\_ssal\_rgb2\_17163 rgb2 Salmo salar cDNA clone ssal\_rgb2\_528\_099\_rev 5', mRNA sequence  
l gi|89837133|gb|DY696483.1|DY696483 EST\_ssal\_rgb2\_52222 ssalrgb2 mixed\_tissue Salmo salar cDNA Salmo salar cDNA clone ssal\_rgb2\_584\_192\_rev 5', mRNA sequence

9 SNPs detected

A B C D E F G H I J K L M N O P Q R S T U V W X Y Z a b c d e f g h i j k l  cosegregation weighted

130 - - - . - - - - A A A A - A A A A - - - . - A - - . . . . . . . . . . . . .   9/9 60.53
131 - - - . - - - - C C C C - C C C C - - - . - C - - . . . . . . . . . . . . .   9/9 60.53
216 A A A . A A A A - - - - A - - - - A A A A A - A A . . . . . . . . . . . . .   9/9 63.16
217 C C C . C C C C - - - - C - - - - C C C C C - C C . . . . . . . . . . . . .   9/9 63.16
218 T T T . T T T T - - - - T - - - - T T T T T - T T . . . . . . . . . . . . .   9/9 63.16
219 T T T . T T T T - - - - T - - - - T T T T T - T T . . . . . . . . . . . . .   9/9 63.16
220 A A A . A A A A - - - - A - - - - A A A A A - A A . . . . . . . . . . . . .   9/9 63.16
221 C C C . C C C C - - - - C - - - - C C C C C - C C . . . . . . . . . . . . .   9/9 63.16
222 A A A . A A A A - - - - A - - - - A A A A A - A A . . . . . . . . . . . . .   9/9 63.16
